# CT angiography-derived three-dimensional pulmonary vein topography is related to the outcome after cryoballoon ablation

**DOI:** 10.3389/fcvm.2025.1496922

**Published:** 2025-05-08

**Authors:** Martin Saal, Annika Esser, Torsten Becker, Florian Stöckigt, Yazan Mohsen, Marc Horlitz, Michael Haude, Dennis Rottländer

**Affiliations:** ^1^ Department of Cardiology, Rheinland Klinikum Neuss, Neuss, Germany; ^2^ Department of Cardiology, Krankenhaus Porz am Rhein, Cologne, Germany; ^3^ Department of Cardiology, University Hospital Bonn, Bonn, Germany; ^4^ Department of Cardiology, Faculty of Health, School of Medicine, University Witten/Herdecke, Witten, Germany

**Keywords:** atrial fibrillation, cryoballoon, pulmonary vein isolation, computer tomography angiography, angulation, pulmonary vein anatomy

## Abstract

**Background:**

Following cryoballoon ablation, 20%–30% of the patients show recurrent atrial fibrillation (AF) in long-term follow-up as a consequence of incomplete circumferential ablation lines. Patient selection using computer tomography angiography (CTA)-derived parameters might be feasible to assign patients for cryoballoon ablation according to pulmonary vein (PV) anatomy and topography.

**Methods:**

We aimed to analyze the impact of anatomical and topographic PV parameters on the procedural outcome of cryoballoon PVI using a retrospective analysis of 106 patients with paroxysmal AF and preprocedural CTA.

**Results:**

Clinical follow-up of the study cohort revealed 78 patients (73.6%, PVI success group) without and 28 patients (26.4%, PVI failure group) with recurrent AF 12 months after cryoablation. Anatomical measurements such as PV diameter, PV area, PV perimeter, or PV eccentricity were not associated with procedural success. The number of occlusion attempts in the right inferior PV was significantly higher in the PVI failure group indicating a technical more complex balloon occlusion. The septum angle *α* (septum–PV) was significantly lower in the superior PVs of the PVI failure group indicating a direct relation of transseptal puncture site to procedural success. Furthermore, orifice angle *β* (PV orifice–PV course) was increased and intra-atrial angle *γ* (septum–PV course) was decreased in the inferior PVs of the PVI failure group.

**Conclusion:**

Patient selection using CTA prior to cryoballoon ablation might influence the procedural success of cryoballoon PVI. While PV anatomy in regard to vein size and shape was not associated with procedural outcome, septum, orifice, and intra-atrial angulation were related to procedural success.

## Introduction

Pulmonary vein isolation (PVI) via cryoballoon ablation represents a standard approach in the interventional management of paroxysmal atrial fibrillation (AF), offering a comparable procedural outcome to radiofrequency (RF) ablation when utilizing 3D mapping on a point-by-point basis (1). However, despite its effectiveness, a notable proportion of patients, ranging from 20% to 30%, experience a recurrence of AF during long-term follow-up (1). This recurrence might be attributed to incomplete lesion lines, often resulting from inadequate balloon occlusion of the pulmonary veins (PVs) (2). Recent studies have highlighted several anatomical and topographical factors as critical determinants of long-term procedural success, including PV ostia size (3–8), shape (3, 7–9), volume (8), and orientation (4, 8–11) and left atrium (LA) size (12). Especially, spatial PV orientation might be an important factor in the procedural outcome of cryoballoon PVI. To date, no standardized assessment of left atrial topographical angulations exists. Accurately measuring these parameters in three-dimensional (3D) geometry might be imperative for improving patient outcomes. Traditional approaches have typically relied on two-dimensional measurements in a standardized LA plane, which do not adequately capture the complex 3D relationships essential for optimal PVI planning (4, 10).

Computer tomography angiography (CTA) offers a promising approach for a detailed assessment of PV and LA anatomy, facilitating the identification of atypical anatomies, such as common PV ostium or accessory PVs, that are linked to poorer clinical outcomes (3).

We aimed to use a CTA tool originally developed for planning left atrial appendage closure procedures to assess critical PV parameters for procedural planning in cryoballoon ablation. Implementing this tool in clinical routine could provide an intuitive workflow to enable the widespread use of anatomical and topographical PV parameters to optimize the procedural planning of PVI via cryoballoon ablation. This approach seeks not only to optimize the planning process but also to enhance AF-free survival rates by ensuring complete ablation lines through ablation strategy selection, directly addressing the anatomical challenges that contribute to AF recurrence post-PVI.

## Materials and methods

### Study participants

For this retrospective analysis, 170 consecutive patients with AF and baseline CTA undergoing PVI using cryoballoon ablation between 1 January 2016 and 31 December 2020 were screened at two German centers, namely, Rheinlandklinikum Neuss and Krankenhaus Porz am Rhein. The study excluded patients with persistent AF; those presenting with accessory PVs or a common PV ostium, inadequate CTA quality, PV stenosis, and situs inversus; and those who experienced procedural complications, such as pericardial tamponade, leading to premature termination of PVI. In persistent AF, atrial fibrosis and substrate abnormalities are more prevalent, making PVI alone less likely to be successful. Consequently, these patients were excluded. A common ostium was defined as a single venous trunk shared by multiple PVs without distinct separation at the ostium level. Moreover, short common ostia with ≤5 mm trunk were excluded to maintain a standardized cohort and ensure reliable outcome comparisons.

The study was conducted in adherence to the Helsinki Declaration and standards of good clinical practice and was approved by the Ethical Committee of the University of Witten–Herdecke (approval number: S-44/2021), ensuring all participants provided written informed consent for the utilization of their data.

**Figure 1:**
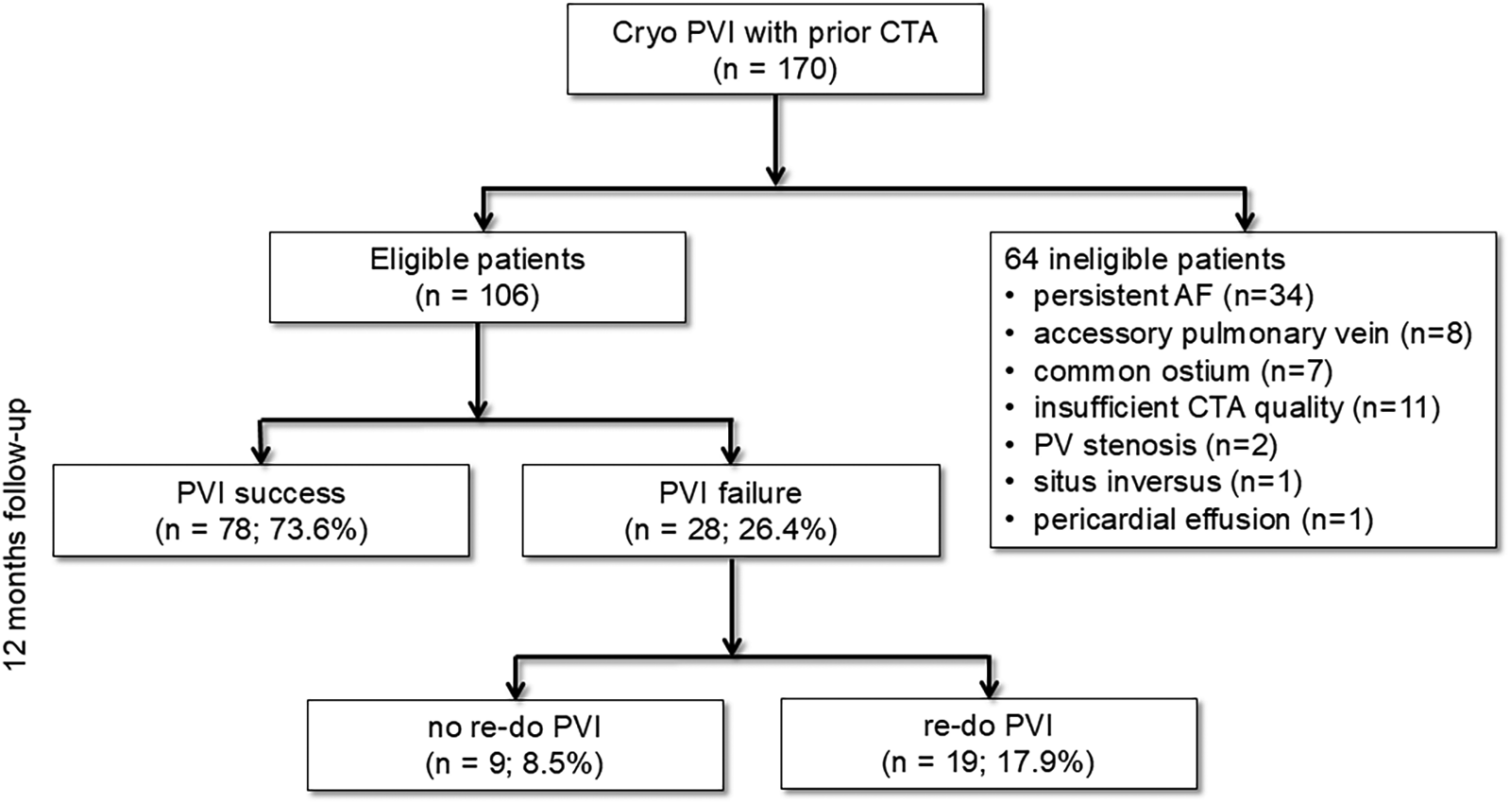
Study flowchart. Cryo PVI, pulmonary vein isolation (PVI) using cryoballoon (Cryo); CTA, computed tomography angiography; PV, pulmonary vein; AF, atrial fibrillation.

Participants were subjected to a 12-month clinical follow-up, which included a 7-day Holter ECG and a structured patient interview at 3 and 12 months, to assess the long-term efficacy of the procedure. To provide a comprehensive overview of the study's design, Figure 1 shows the study flow diagram. Of note, antiarrhythmic medication was discontinued immediately after the PVI. AF recurrence was defined as the documented recurrence of AF or atrial tachycardia lasting longer than 30 s, as previously reported (1).

### Pulmonary vein isolation using cryoballoon

PVI for paroxysmal AF using the cryoballoon was performed under deep sedation using midazolam, fentanyl, and propofol. Right and/or left femoral veins were used as vascular access for a right ventricular quadripolar and a decapolar deflectable coronary sinus catheter. The decapolar deflectable catheter was also used for phrenic nerve pacing during ablation of all right-sided PVs. Phrenic nerve capture was monitored by continuous palpation and/or observation of the compound motor action potential on the surface ECG. The transseptal puncture was guided by fluoroscopy. Afterward, activated clotting time was kept at a target of >300 s using intravenous heparin. After transseptal puncture, a steerable 15 French diameter sheath (FlexCath Advance, Medtronic, Minneapolis, USA) was placed in the LA. Throughout the procedure, it was continuously flushed with heparinized saline. Only the 28 mm cryoballoon (Arctic Front Advance Pro, Medtronic, Minneapolis, USA) was used in this study. PV signals during the procedure were assessed by a 20 mm octapolar inner lumen spiral mapping catheter (Achieve, Medtronic, Minneapolis, USA). PVs were isolated clockwise from the left superior to the right superior. After obtaining PV occlusion—assessed by injection of contrast agent—by optimal alignment of the sheath, the cryoballoon, and the PV, freezing cycles with a standard duration of 180–240 s were used (depending on time to effect). Procedural success was defined as the elimination of all PV potentials and exit block (pacing inside PV) on the spiral mapping catheter.

### Occlusion grading

All fluoroscopy imaging of the index procedure (PVI) was retrospectively analyzed by two independent experts (MS, DR). The number of occlusion attempts was counted per PV. Furthermore, occlusion grading (OG) was performed as previously described (12):

OG1: poor occlusion, massive leakage, PV shape cannot be confirmed.
OG2: incomplete occlusion, PV shape can be confirmed.
OG3: complete occlusion.

### Preprocedural CT angiography

All patients underwent preprocedural CTA for evaluation of LA anatomy. For CTA a 256-slice Brilliance iCT scanner (Philips Healthcare, Amsterdam, Netherlands) was used in accordance with the Society of Cardiovascular Computed Tomography (SCCT) guidelines ( 13 ). A tube current between 200 and 360 mAs at 120 kV, adjusting primarily the mAs based on body habitus. CTA was preferably performed in ECG-gated step and shoot technique. The collimation of CTA was 256*0.6 mm, and the rotation time was 0.27 s. Prior to the scanning metoprolol was administered only, if necessary (50–150 mg orally 1 h before CT scan or 5–25 mg intravenously during the scan), aiming for a heart rate <65 beats/min. A contrast agent (Imeron 350, Bracco, Milano, Italy) was administered with a volume of 80 ml (5.0 ml·s-1) followed immediately by a 50 ml saline chaser. Data were reconstructed at 75% of the R–R interval, with a slice thickness of 0.5 mm and a reconstruction interval of 0.3 mm.

### CTA analysis

**Figure 2:**
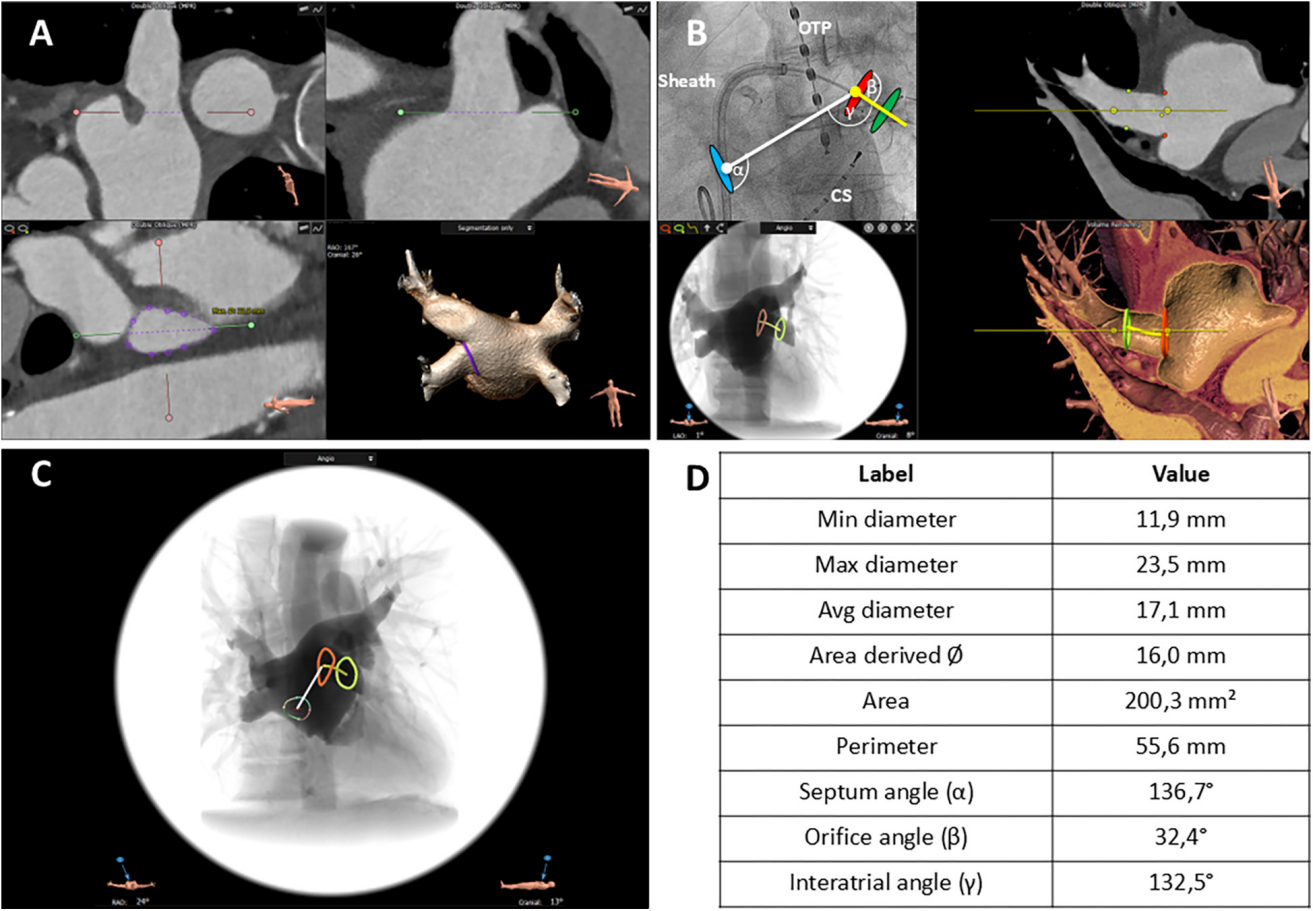
CTA-derived workflow to assess pulmonary vein (PV) anatomy and topography. **(A)** Definition of the ostial pulmonary vein (PV) diameter in two perpendicular planes. The red and green lines mark the planes, and the dotted lines delineate the particular PV diameter in each orientation (upper left, upper right). Automatic border detection of the PV orifice in a 2D planar orientation; the purple line encircles the orifice (lower left). 3D reconstruction of the left atrium from a posterior view; the purple line corresponds to the encircled PV orifice in 3D orientation (lower right. **(B)** Angiographic view (left anterior oblique 50°) illustrating the relative position of the PV ostium, site of transseptal puncture, catheter sheath, and cryoballoon (upper left). 2D planar view of the left atrium and proximal course of the left inferior PV; the red dots mark the orifice area, and the green dots mark the PV area in the further course (upper right). Simulated angiographic view (frontal) of the orifice and proximal course of the left inferior PV computed from the CT angiography (lower left). 3D reconstruction of the upper right two-dimensional view (lower right). Blue oval, puncture site of the interatrial septum; red oval, ostium of the left inferior PV; green oval, area in the further course of the PV. *α* angle, interatrial septum—PV ostium. *β* angle, PV ostium—PV course. *γ* angle, interatrial septum—PV course. OPT, oral temperature probe; CS, catheter in the coronary sinus. White line, distance interatrial septum—PV ostium. Yellow line, course of the PV starting at the PV ostium. **(C)** Simulated angiographic view (frontal) comprising all information including intra-atrial septum, PV ostium (red line), and course of PV (green line). Varicolored circle with central red crosshair: puncture site of the interatrial septum. White line: distance interatrial septum to PV ostium. Yellow line: further course of the PV. **(D)** Measurements of the anatomic characteristics of PV ostium and relevant angles.

The 3mensio Structural Heart (prototype version 10.2) software (Pie Medical Imaging, Maastricht, Netherlands) analyzes the left atrium using multiplanar reconstructions and volume rendering techniques, as previously described, and is commercially used for planning left atrial appendage closure procedures (14). Figure 2 shows the algorithm of the CTA analysis in a stepwise approach. In brief, after importing CTA images as Digital Imaging and Communications in Medicine (DICOM) files in the 3mensio software, the left atrial appendage workflow was used for PV analysis. The algorithm started with automated 3D reconstruction of the left atrium, followed by defining the ostial PV diameters in two perpendicular planes. Automatic border detection in 2D planar orientation was used to determine PV orifices. All measurements were proofed and if necessary corrected by two independent imaging specialists (MS, AE). Automatic measurements of minimal diameter, maximum diameter, average diameter, area-derived diameter, perimeter-derived diameter, ostial area, and ostial perimeter were performed by the software and proofed by the imaging specialists (Supplementary Figure S1). Eccentricity, defined as the ratio of the distances of a point on the ellipse from the focus and the directrix, was automatically calculated by the 3mensio software (Supplementary Figure S1). To further characterize the course of the PVs, a second measurement was performed 10 mm distal to the PV orifice. In early branching PVs, the imaging specialists chose the branch where the mapping catheter was placed during the procedure. Following the delineation and measurement of the PV ostia, the site for transseptal puncture was identified utilizing the corresponding 3mensio workflow, as outlined in previous publications (14, 15). The transseptal puncture site was estimated based on its typical location within the fossa ovalis, as the exact site could not be retrospectively determined. This assumption aligns with standard procedural practice to ensure optimal catheter maneuverability. To investigate the anatomic conditions for perfect PV occlusion with the cryoballoon, the following vector angles were measured: septum angle (*α*), septum to PV ostium; orifice angle (*β*), angle between PV ostium and course of PV (10 mm from PV ostium); intra-atrial angle (*γ*), angle between distance; interatrial septum to PV ostium and distance, PV ostium to PV course. All angles were automatically calculated by the software and checked by both imaging specialists. The placement of the mapping catheter in cases of early branching pulmonary veins was determined by correlating procedural fluoroscopy with CTA-derived PV anatomy.

### Statistical analysis

Statistical analysis was performed using PASW Statistics 18 software (SPSS, Chicago, USA). All variables were tested for normal distribution with the Kolmogorov–Smirnov test. In the case of normal distribution, the results are given as mean ± standard deviation (SD) or ± standard error (SEM) as indicated, otherwise as median and 95% confidence interval. Differences between groups and subgroups were evaluated by chi-square test for discrete variables and student *t*-test or one-way ANOVA with Scheffe post hoc testing for continuous variables. For ordinal data, the Mann–Whitney *U*-test was used. ROC curve analysis including Youden index assessment was performed using MedCalc statistical software 18.10 (MedCalc Software, Ostend, Belgium). *p* < 0.05 was considered statistically significant.

## Results

CTA identified 15 patients (8.8% of the total cohort, *n* = 170) with atypical PV anatomy, who were excluded from the study (Figure 1). Additionally, 34 patients with persistent AF, 11 patients with insufficient CTA imaging quality, 2 patients with PV stenosis, and 1 patient with situs inversus were also excluded from the analysis (Figure 1). In terms of procedural outcomes, 78 of 106 patients (73.6%) had no recurrent AF in 12 months following PVI and were allocated to the PVI success group. Conversely, PVI failure was found in 28 patients (26.4%). In the overall cohort, 55.7% (*n* = 59) of the patients were male, and the mean age was 63.1 ± 1.0 years (*n* = 106). However, the baseline characteristics of both groups were well balanced without significant differences (Table 1).

**Table 1:** Baseline characteristics.

Parameters	PVI success		PVI failure		*p*-value
	*n*	Mean ± SEM or %	*n*	Mean ± SEM or %	
Age (years)	78	63.29 ± 1.19	28	62.61 ± 1.82	0.76
Male	43	55.1	16	57.1	0.86
Patients history					
Arterial hypertension	52	66.67	20	71.43	0.65
Hyperlipidemia	26	33.33	11	39.29	0.57
Obesity	19	24.36	5	17.86	0.49
Family history CVD	13	16.67	2	7.14	0.2
Diabetes mellitus	9	11.54	2	7.14	0.52
History of smoking	10	12.82	3	10.71	0.72
Current smoking	9	11.54	3	10.71	0.86
Coronary artery disease	11	14.1	1	3.57	0.13
CHA_2_DS_2_-VASc score	78	2.22 ± 0.17	28	1.93 ± 0.22	0.93
Echocardiography					
LVEF > 50%	72	92.31	28	100	0.13
LVEF 40%–49%	3	3.85	0	0	0.55
LVEF < 39%	3	3.85	0	0	0.3
LA volume (ml/m^2^)	71	28.62 ± 1.08	26	30.19 ± 1.55	0.44
Medication					
Beta-blocker	52	66.67	20	71.43	0.79
Flecainid	6	7.69	3	10.71	0.66
Dronedarone	2	2.56	3	10.71	0.09
Amiodaron	2	2.56	0	0.0	0.39
Phenprocoumon	9	11.54	3	31.71	0.03
Apixaban	20	25.64	4	14.29	0.19
Dabigatran	1	1.28	2	7.14	0.12
Edoxaban	5	6.41	9	3.57	0.56
Rivaroxaban	35	44.87	16	57.14	0.08
Clinical presentation					
EHRA I	0	0.0	0	0.0	-
EHRA II	16	20.51	2	7.14	0.11
EHRA III	60	76.92	26	92.86	0.07
EHRA IV	2	2.56	0	0.0	0.4

CVD, cardiovascular disease; LVEF, left ventricular ejection fraction; LA, left atrial; EHRA, European Heart Rhythm Association.

All of the 106 included patients had successful PVI using the cryoballoon. One patient was excluded due to pericardial tamponade after isolation of the left superior pulmonary vein with consecutive pericardiocentesis resulting and the termination of the procedure before isolating all PVs. Phrenic nerve injury did not occur in our patient cohort. Procedural characteristics, including procedure time, the volume of contrast agent used, the number of cryoballoon applications, the duration of applications, the lowest balloon temperature, and time to effect (PV isolation) for all PVs (LSPV, LIPV, RSPV, RIPV), showed no significant differences between the success and failure groups ( Table 2 ).

**Table 2:** Procedural characteristics.

Parameters	PVI success	PVI failure	*p*-value
	Mean ± SEM or % *n* = 78	Mean ± SEM or % *n* = 28	
Contrast agent (ml)	100.74 ± 4.06	114.86 ± 7.33	0.12
Procedure time (min)	74.62 ± 3.44	81.54 ± 5.02	0.64
Procedural data LSPV			
Number of applications	1.40 ± 0.07	1.43 ± 0.16	0.73
Total duration of cryo-application (s)	308.00 ± 18.95	324.39 ± 35.39	0.94
Lowest balloon temperature (°C)	−50.62 ± 0.62	−50.78 ± 2.71	0.65
Time to effect (s)	61.96 ± 4.14 (*n* = 53)	56.83 ± 5.37 (*n* = 24)	0.45
Lowest esophageal temperature (°C)	33.32 ± 0.44	33.21 ± 1.39	0.29
Procedural data LIPV			
Number of applications	1.07 ± 0.03	1.29 ± 0.12	0.23
Total duration of cryo-application (s)	244.44 ± 7.10	302.21 ± 25.54	0.06
Lowest balloon temperature (°C)	−39.18 ± 2.76	−45.71 ± 1.85	0.25
Time to effect (s)	39.18 ± 2.76 (*n* = 55)	49.24 ± 5.30 (*n* = 20)	0.62
Lowest esophageal temperature (°C)	40.48 ± 2.83	30.91 ± 1.94	0.42
Procedural data RSPV			
Number of applications	1.07 ± 0.03	1.25 ± 0.10	0.33
Total duration of cryo-application (s)	241.33 ± 7.95	281.07 ± 20.95	0.24
Lowest balloon temperature (°C)	−50.91 ± 0.60	−50.18 ± 2.17	0.43
Time to effect (s)	56.91 ± 4.10	47.96 ± 5.23	0.94
Lowest esophageal temperature (°C)	33.87 ± 0.72 (*n* = 61)	35.19 ± 1.24 (*n* = 16)	0.43
Procedural data RIPV			
Number of applications	1.18 ± 0.06	1.32 ± 0.14	0.15
Total duration of cryo-application (s)	269.07 ± 13.01	305.21 ± 25.64	0.07
Lowest balloon temperature (°C)	−48.04 ± 0.67	−46.68 ± 2.06	0.19
Time to effect (s)	69.54 ± 4.59 (*n* = 46)	59.69 ± 6.74 (*n* = 13)	0.87
Lowest esophageal temperature (°C)	30.68 ± 1.02	31.57 ± 1.89	0.91

LSPV, left superior pulmonary vein; LIPV, left inferior pulmonary vein; RSPV, right superior pulmonary vein; RIPV, right inferior pulmonary vein.

PV ostia area, diameter, perimeter, and eccentricity were not different between PVI success and PVI failure (Table 3). LIPV revealed the smallest diameter, area, and perimeter, while RSPV showed the largest PV dimensions (Table 3). Eccentricity was lowest for the RIPV indicating the ostium being close to a regular circle (PVI success: 0.14 ± 0.01 vs. PVI failure: 0.16 ± 0.01; *p* = 0.33). The highest eccentricity was found for LSPV (PVI success: 0.29 ± 0.01 vs. PVI failure: 0.30 ± 0.02; *p* = 0.83).

**Table 3:** Measurements of PV ostia using 3D reconstructions of CT angiographies.

Parameters	PVI success	PVI failure	*p*-value
	Mean ± SEM *n* = 78	Mean ± SEM *n* = 28	
LSPV ostium			
Minimal diameter (mm)	18.3 ± 0.54	17.65 ± 0.72	0.50
Maximal diameter (mm)	25.8 ± 0.49	25 ± 0.59	0.37
Average diameter (mm)	22.06 ± 0.48	21.33 ± 0.60	0.40
Area-derived diameter (mm)	21.75 ± 0.49	21.1 ± 0.60	0.47
Area (mm²)	385.14 ± 17.71	357.18 ± 20.30	0.37
Perimeter (mm)	70.28 ± 1.59	68.71 ± 1.84	0.59
Eccentricity	0.29 ± 0.01	0.30 ± 0.02	0.83
LIPV ostium			
Minimal diameter (mm)	15.72 ± 0.36	14.97 ± 0.53	0.27
Maximal diameter (mm)	21.66 ± 0.35	21.1 ± 0.58	0.42
Average diameter (mm)	18.69 ± 0.32	18.04 ± 0.50	0.29
Area-derived diameter (mm)	18.50 ± 0.33	17.86 ± 0.52	0.31
Area (mm²)	275.34 ± 10.14	256.29 ± 15.58	0.33
Perimeter (mm)	60.01 ± 1.0	58.18 ± 1.61	0.35
Eccentricity	0.27 ± 0.01	0.28 ± 0.02	0.68
RSPV ostium			
Minimal diameter (mm)	21.09 ± 0.38	20.65 ± 0.60	0.54
Maximal diameter (mm)	26.33 ± 0.35	26.08 ± 0.67	0.72
Average diameter (mm)	23.71 ± 0.33	23.35 ± 0.59	0.58
Area-derived diameter (mm)	23.57 ± 0.34	23.34 ± 0.61	0.74
Area (mm²)	435.53 ± 13.56	434.65 ± 22.74	0.97
Perimeter (mm)	82.24 ± 7.20	74.6 ± 1.93	0.53
Eccentricity	0.20 ± 0.01	0.20 ± 0.02	0.93
RIPV ostium			
Minimal diameter (mm)	20.37 ± 0.42	19.72 ± 0.77	0.44
Maximal diameter (mm)	23.58 ± 0.41	23.41 ± 0.75	0.83
Average diameter (mm)	21.99 ± 0.40	21.62 ± 0.75	0.64
Area-derived diameter (mm)	21.89 ± 0.40	21.51 ± 0.75	0.65
Area (mm²)	385.5 ± 14.01	375.39 ± 25.89	0.72
Perimeter (mm)	69.37 ± 1.26	68.28 ± 2.33	0.67
Eccentricity	0.14 ± 0.01	0.16 ± 0.01	0.33

LSPV, left superior pulmonary vein; LIPV, left inferior pulmonary vein; RSPV, right superior pulmonary vein; RIPV, right inferior pulmonary vein; CT, computed tomography.

A small number of PVs in both groups revealed a maximum diameter of >28 mm (PVI success: RIPV 10.3%, RSPV: 25.6%, LIPV: 1.2%, LSPV: 29.5%; PVI failure: RIPV 14.3%, RSPV: 28.6%, LIPV: 0.0%, LSPV: 14.3%) and thus exceeding the cryoballoon diameter of 28 mm.

**Figure 3:**
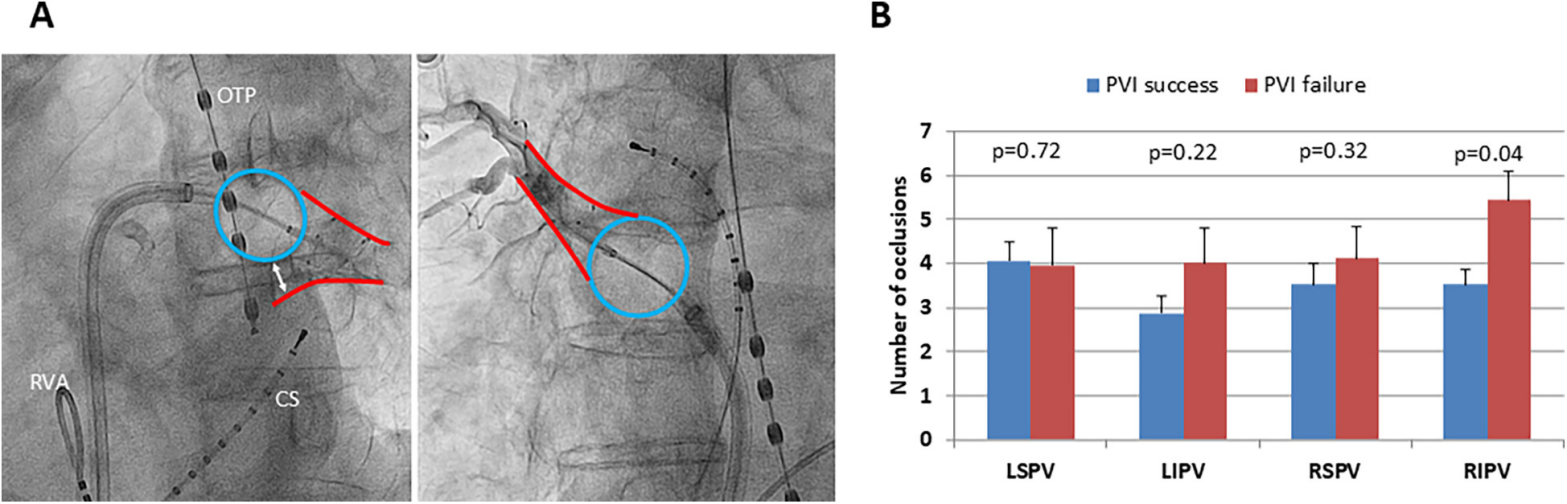
Number of occlusions and freedom from AF recurrence following PVI. **(A)** Left side: x-ray (LAO 50°) of occlusion grade 1 with significant reflow of contrast agent due to poor balloon occlusion of the left inferior pulmonary vein. Right side: x-ray (RAO 30°) of occlusion grade 3 with no reflow of contrast agent due to optimal balloon occlusion of the right superior pulmonary vein. Blue circle: inflated cryoballoon, the red outlines mark the outer borders of the particular pulmonary vein, and the white double arrow marks the extent of poor occlusion. OTP, esophageal temperature probe; CS, coronary sinus catheter; RVA, right ventricular apex catheter. **(B)** Mean amount of occlusion attempts for each pulmonary vein to reach optimal occlusion grading, distinguished between successful pulmonary vein isolation (PVI success, blue bars) and AF recurrence (PVI failure, red bars) 1 year following index procedure; the black lines mark standard error of the mean. LSPV, left superior pulmonary vein; LIPV, left inferior pulmonary vein; RSPV, right superior pulmonary vein; RIPV, right inferior pulmonary vein. *p < 0.05.

Occlusion grading for all PVs was performed (Figure 3A). No difference in occlusion grading was found between the groups (PVI success: LSPV 2.8 ± 0.05, LIPV 2.9 ± 0.03, RSPV 2.7 ± 0.06, RIPV 2.6 ± 0.06 vs. PVI failure: LSPV 2.8 ± 0.08, LIPV 2.9 ± 0.04, RSPV 2.8 ± 0.08, RIPV 2.7 ± 0.09; *p* = 0.41, *p* = 0.28, *p* = 0.43, *p* = 0.64). For RIPV, significantly more numbers of occlusion attempts before freezing were needed in the PVI failure group indicating a more complex balloon occlusion (*p* = 0.04; Figure 3B).

**Figure 4:**
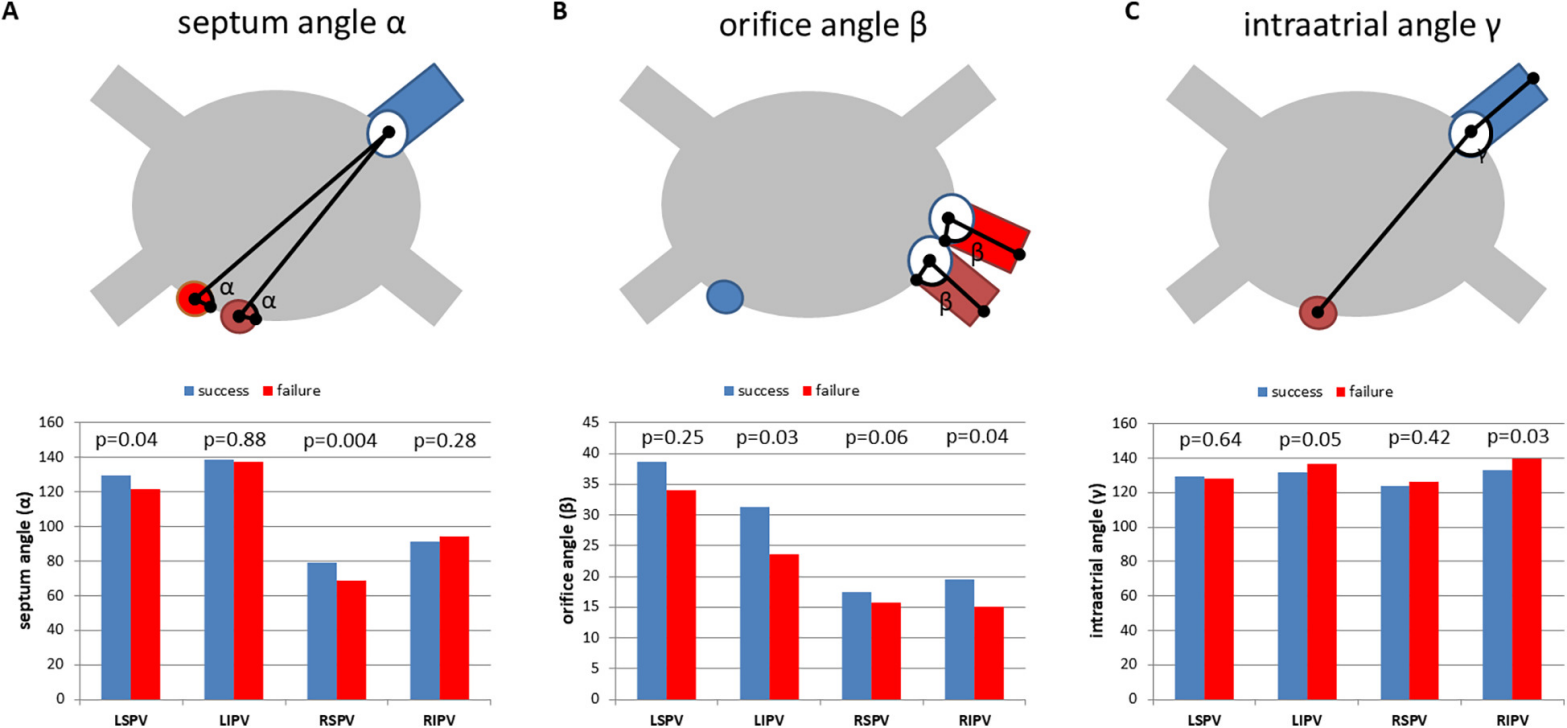
One-year freedom of AF is associated with PVI topography. **(A)** Mean septum angle (angle *α*), measured between the intra-atrial septum (two different positions: red and brown) and the PV orifice (PV marked blue). PVI success: no recurrent AF 1 year after PVI using the cryoballoon (blue bars). PVI failure: recurrent AF within the first year after ablation (red bars). **(B)** Mean orifice angle (angle *β*), measured between the PV orifice and the course of the pulmonary vein. Blue: transseptal puncture site. Red and brown: two different PV orientations indicating the topographical variation of orifice angle *β*. **(C)** Mean intra-atrial angle (angle *γ*) measured between the distance of the interatrial septum (brown) to the PV ostium and the distance of the PV ostium to the PV course. Blue: PV. PVI success (freedom from AF within 1 year, blue bars) and PVI failure (recurrent AF within the first year after PVI, red bars). LSPV, left superior pulmonary vein; LIPV, left inferior pulmonary vein; RSPV, right superior pulmonary vein; RIPV, right inferior pulmonary vein. Mean and SEM. *p < 0.05.

Septum angle *α*, which displays the angle between the septum and the PV ostium was significantly higher for the superior PVs (LSPV and RSPV) in the PVI success group compared with the PVI failure group (LSPV: *p* = 0.04, RSPV: *p* = 0.004; Figure 4A). Furthermore, orifice angle *β*, characterizing the angle between the PV ostium and the course of the PV, was significantly lower for LIPV and RIPV in the PVI failure group (LIPV: *p* = 0.03, RIPV: *p* = 0.04; Figure 4B). Of note, the inferior PVs revealed a significantly lower intra-atrial angle *γ*, which represents the angle between the connecting line of the interatrial septum to the PV ostium and the connecting line of the PV ostium to the PV course (LIPV: *p* = 0.05, RIPV: *p* = 0.03; Figure 4C).

**Figure 5:**
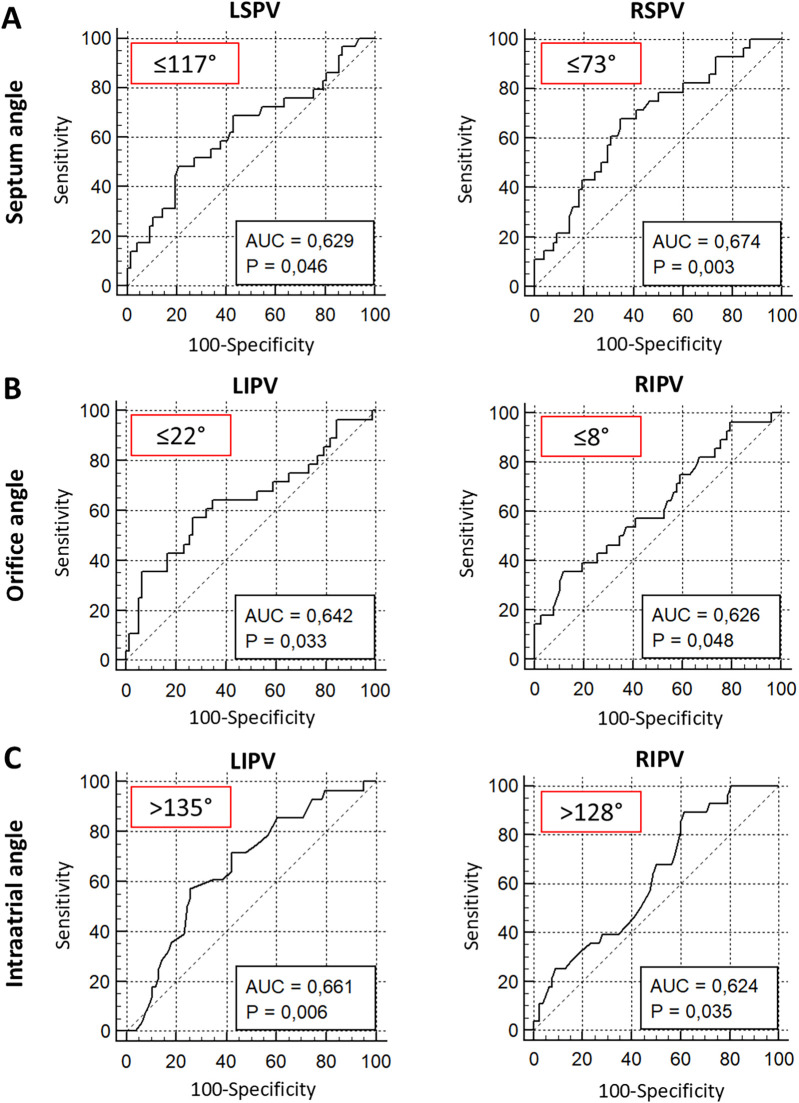
ROC analysis of septum, orifice, and intra-atrial angulation predicting 1-year freedom from AF after PVI using the cryoballoon. ROC analysis of septum angle *α* **(A)**, orifice angle *β* **(B)**, and intra-atrial angle *γ* **(C)** for prediction of 1-year freedom from AF. Red box: optimal threshold (Youden index). AUC, area under the curve; LSPV, left superior pulmonary vein; LIPV, left inferior pulmonary vein; RSPV, right superior pulmonary vein; RIPV, right inferior pulmonary vein.

To further evaluate the impact of the angles *α*, *β*, and *γ* on the procedural success of PVI using the cryoballoon ROC analyses were performed. For the septum angle *α* of both superior veins, ROC analysis revealed a favorable angle of ≤117° (LSPV, *p* = 0.046) and ≤73° (RSPV, *p* = 0.003) to maintain procedural success defined as freedom of AF in the first 12 months (Figure 5A). Furthermore, an orifice angle *β* ≤ 22° (LIPV, *p* = 0.033) and ≤8° (RIPV, *p* = 0.048) indicated the procedural success of cryoballoon PVI (Figure 5B). The intra-atrial angle *γ* was related to the freedom of AF after PVI, when the inferior PVs had an angle of >135° (RIPV, *p* = 0.006) and >128° (LIPV, *p* = 0.035) (Figure 5C). The other ROC analyses revealed no significant angulations related to the three angles (Supplementary Figure S2).

In 19 patients with PVI failure, re-PVI was performed using 3D mapping and radiofrequency ablation (19/26, 73.1%). Reconnection was found in 33 PVs, and corresponding unfavorable angulations could be confirmed in 29 PVs (87.9%, Table 4).

**Table 4:** Pulmonary vein topography and reconnected pulmonary veins in 3D mapping in patients with redo PVI.

	RSPV			RIPV			LSPV			LIPV				
PVI failure #	Orifice angle (°)	Septum angle (°)	Intra-atrial angle (°)	Orifice angle (°)	Septum angle (°)	Intra-atrial angle (°)	Orifice angle (°)	Septum angle (°)	Intra-atrial angle (°)	Orifice angle (°)	Septum angle (°)	Intra-atrial angle (°)	Reconnected PV	Favorable angle
**3**	14.7	74	135	8.1	97.2	130	26.3	116.5	119	12.2	134.7	132	RSPV	SA ≤ 73°
**4**	15	60.7	137	9.8	89.5	148	3.9	127.1	133	1.4	143.7	145	LIPV RSPV RIPV	OA ≤ 22°, IA > 135° SA ≤ 73° OA < 8°, IA > 128°
**5**	14.1	64.7	131	10.8	84.6	120	16.2	123.6	124	21.8	158.3	144	RSPV	SA ≤ 73°
**8**	20.3	74	121	27.8	100.9	119	42.2	141.8	143	41.1	147.2	155	RSPV RIPV	SA ≤ 73° OA < 8°, IA > 128°
**9**	14.7	43.5	113	7.4	84	129	51.9	151.2	126	14.9	143.4	139	LSPV	OA ≤ 22°, IA > 135°
**10**	13.8	75.8	122	12.8	111.4	161	17.7	84.3	101	9	135	139	RSPV	SA ≤ 73°
**11**	19.2	85	128	26.5	111.2	138	36.4	135	129	37.6	137.9	128	LSPV RIPV	SA ≤ 117° OA < 8°, IA > 128°
**12**	1.9	65.6	130	1.8	73.7	150	36.6	146.1	132	46.1	151.9	123	RSPV LIPV	SA ≤ 73° OA ≤ 22°, IA > 135°
**13**	4.2	74.5	127	9.3	87.3	140	38.3	127	121	40.7	158.9	126	LSPV RIPV	SA ≤ 117° OA < 8°, IA > 128°
**14**	9.4	61.1	132	7.2	106	154	39.3	131.6	127	25.4	148	135	LIPV LSPV	OA ≤ 22°, IA > 135° SA ≤ 117°
**15**	9.1	75	112	14.1	97.8	143	30.5	100.9	131	3.3	124.4	142	RIPV RSPV	OA < 8°, IA > 128° SA ≤ 73°
**17**	18.3	59	124	20.5	105.2	131	33.3	105.7	138	10.7	138.3	117	LIPV RSPV	OA ≤ 22°, IA > 135° SA ≤ 73°
**19**	4.6	77.7	140	18.4	91.7	131	6.6	114.4	140	10.5	144	156	RSPV RIPV	SA ≤ 73° OA < 8°, IA > 128°
**21**	7.9	61	131	3.1	68.4	146	49.8	145.1	117	11.6	140.3	133	RIPV	OA < 8°, IA > 128°
**22**	41.9	96.6	117	39.1	89.4	151	25.7	140.7	118	45.1	143.6	132	RIPV	OA < 8°, IA > 128°
**23**	0.7	97.4	122	28.5	73.8	159	65.7	151.5	122	31.4	131.1	129	LSPV RIPV	SA ≤ 117° OA < 8°, IA > 128°
**26**	3.6	74	94	22.5	119.7	123	59.9	107.5	124	34.7	148.1	146	RSPV LIPV	SA ≤ 73° OA ≤ 22°, IA > 135°
**27**	28.6	75	135	27	94.5	137	51.6	119.3	139	65.7	17.4	128	LSPV RSPV RIPV	SA ≤ 117° SA ≤ 73° OA < 8°, IA > 128°
**28**	23.8	81.2	120	11.3	97	136	68.3	145.5	126	38.7	145.7	140	LIPV	OA ≤ 22°, IA > 135°

LSPV, left superior pulmonary vein; LIPV, left inferior pulmonary vein; RSPV, right superior pulmonary vein; RIPV, right inferior pulmonary vein; SA, septum angle; OA, orifice angle; IA, intra-atrial angle. Green, correct prediction of PV reconnection by PV topography; red, incorrect prediction of PV reconnection by PV topography.

The bold numbers are the individual patients numbers of the PVI failure group.

## Discussion

PVI is a standard technique in the treatment of paroxysmal AF (16). While the Arctic Front Advance cryoballoon is a well-established single-shot device, other balloon-based technologies such as laser balloons, RF balloons, other cryoballoon systems, or balloon-supported pulsed-field ablation (PFA) are emerging in the field of electrophysiology (1, 17, 18). All balloon-based PVI systems warrant perfect PV occlusion indicating circumferential wall contact for complete ablation lines and procedural success. Previous publications reported on the association of LA and PV anatomy and the outcome of PVI using the cryoballoon (3–12). However, the results varied between the studies, no standardized approach was used and mostly small patient cohorts were included. Furthermore, the 3D anatomy and spatial orientation of the PVs and LA were not taken into consideration. We aimed to analyze the impact of anatomical and topographical parameters on procedural success (freedom of AF recurrence) using 3D reconstructions from left atrial CTAs combined with a standardized analysis algorithm provided by the 3mensio structural heart software. Our main results are as follows:

(1) Anatomical measurements such as PV diameter, PV area, PV perimeter, or PV eccentricity were not associated with 1-year freedom from AF following cryoballoon PVI.
(2) The number of occlusion attempts was significantly increased for the right inferior PV in the PVI failure group, while occlusion grading was comparable for all PVs in both groups.
(3) The spatial orientation of the PVs was directly related to procedural success. In PVI failure, septum angle *α* was elevated for the superior PVs, orifice angle *β* was significantly reduced for the inferior PVs, and intra-atrial angle *γ* was significantly lower for the inferior PVs.
(4) ROC analysis revealed thresholds for septum angle *α*, orifice angle *β*, and intra-atrial angle *γ* directly related to long-term freedom from AF following PVI using the cryoballoon.

### Comparison to controlled randomized trials

In our cohort of patients with paroxysmal AF undergoing cryoballoon PVI, 73.6% had freedom from AF in the first 12 months following the index procedure. The procedural success rate was in the range of cryoballoon landmark trials (Fire and Ice, STOP-AF, Cryo-First, Early AF), where the 1-year freedom from AF rate varies between 67.8% and 82.2% (1, 18–20). Since freedom from AF highly depends on the methods used for AF screening, in this study 7-day Holter ECG and structured patient interviews were used in the follow-up visits. Otherwise, the baseline characteristics of the two study groups (PVI success, PVI failure) were well balanced excluding any influence on the results of the procedural success. Of note, for all PVs procedure time, amount of contrast agent, total number of cryoballoon applications, duration of cryoballoon applications, lowest balloon temperature, and time to effect (PV isolation) were not significantly different between both groups. These results indicate comparable procedural characteristics. Of note, conventional parameters such as time to effect or balloon temperature did not differ in both groups and were no predictors of long-term procedural success in this cohort of paroxysmal AF.

### PV anatomy and balloon occlusion

While for RF ablation in a point-by-point manner, contact force measurements are well established, these technologies are missing for single-shot PVI devices such as the cryoballoon ( 21 ). One might speculate that angiographic evaluation of balloon occlusion grade helps predict complete circumferential ablation of the PVs and therefore freedom from postprocedural AF. However, no difference in occlusion grading was observed between PVI success and PVI failure. Our results are in line with a previous study, which showed that a complete PV occlusion is not associated with procedural success for all PVs except RIPV ( 12 ). For RIPV, we found significantly more numbers of occlusion attempts prior to cryotherapy indicating a technically more challenging positioning of the cryoballoon to achieve complete occlusion. A previous study found RIPV as the most common location of PV reconnection following PVI using the cryoballoon ( 22 ). This finding implies that anatomical and topographical factors could prevent complete balloon occlusion and therefore isolation of RIPV by the cryoballoon.

### Impact of pulmonary vein anatomy and topography on PVI outcomes

Previous studies revealed PV ostia size (3–8), shape (3, 7–9), and volume (8) and LA size (12) as important factors of long-term procedural efficacy and freedom from AF recurrence. Our results using a standardized 3D approach could not confirm these previous results as there was no difference in anatomical PV parameters between PVI success and PVI failure. Of note, a standardized, semiautomated approach supported by 3D reconstructions was used to rule out incorrect measurements. Furthermore, several anatomical parameters such as perimeter, area, diameter, or eccentricity were analyzed, making the results more reliable. Measurements using the 3mensio structural heart software are validated in several studies for structural heart interventions (22, 23).

Several other factors may explain differences between our and previous studies, including study design, statistical power, measurement techniques, and the specific outcomes assessed. Prior studies have examined the role of PV anatomy with varying methodologies and endpoints. For example, Kurokawa et al. investigated the optimal PV ostium diameter for cryoballoon ablation in 71 patients but only assessed nadir PV temperature as an endpoint rather than long-term procedural success, limiting its applicability to our findings ( 7 ). Similarly, studies by Chen et al. ( 3 ) (32 patients) and Sorgente et al. ( 9 ) (52 patients) included smaller patient cohorts, which may not provide sufficient statistical power to generalize findings regarding PV eccentricity. Furthermore, Matsumoto et al. ( 8 ), in a study of 100 patients, found no significant effect of PV eccentricity on incomplete cryoballoon ablation, supporting our findings.

While PV anatomy was not related to PVI success, the spatial orientation of the PVs was associated with the outcome following cryoballoon therapy in paroxysmal AF. One might speculate that correct balloon alignment is most important for the resulting contact force and complete ablation lines. While the local temperature at the ostial circumference of the PVs could not be monitored during the procedure, angulation of the cryoballoon catheter could be controlled and regulated. We found an increased septum angle *α* for the superior PVs in the PVI success group, indicating a direct relation to transseptal puncture. We defined an angulation of ≤117° for LSPV and ≤73° for RSPV as a predictor of PVI success. Therefore, preprocedural planning of transseptal puncture might overcome limitations in cryoballoon therapy of the superior PVs. These results are in line with previous findings for left atrial appendage (LAA) closure, where the angle between the septum and LAA predicts complex procedures (15). Furthermore, orifice angle *β*, characterizing the angle between the PV ostium and the course of the PV, was significantly lower for LIPV and RIPV in the PVI failure group. Orifice angle *β* could be modestly influenced by the placement of the mapping catheter in the inferior branch of the inferior PVs. This is in particular important to maintain optimal contact force to the PV ostium. Finally, the inferior PVs revealed a significantly lower intra-atrial angle *γ*, which represents the angle between the connecting line of the interatrial septum to the PV ostium and the connecting line of the PV ostium to the PV course. This angle is directly related to the flexion of the sheath and could be influenced by the steering maneuvers to a certain degree as well as applying more pressure. The intra-atrial angle *γ* was related to freedom from AF after PVI, when the inferior PVs had an angle of >135° (RIPV) and >128° (LIPV). Lower angulations assessed by preprocedural CTA could influence patient selection and 3D mapping combined with RF ablation or pulsed-field ablation (PFA) might be more beneficial for these patients.

### PV reconnection following cryoballoon PVI

Our study indicates that in the majority of cases, reconnected PVs exhibited unfavorable anatomical angulations, particularly in relation to the identified cutoff values predictive of reconnection. For RSPV, a septum angle *α* ≤ 73° was associated with PVI success, confirming that a steeper angle at the transseptal puncture site may contribute to optimal cryoballoon positioning. Similarly, reconnection in the RIPV was frequently observed in veins where the orifice angle *β* was >8° and the intra-atrial angle *γ* was <128°, suggesting that the inferior pulmonary veins with a more acute take-off from the left atrium are at higher risk of incomplete ablation. Furthermore, LSPV and LIPV demonstrated a significant correlation between reconnection and threshold-exceeding angulations. Specifically, LSPVs with a septum angle *α* > 117° and LIPVs with an orifice angle *β* ≥ 22° and intra-atrial angle *γ* < 135° were more prone to reconnection, indicating that unfavorable PV orientation impacts long-term PVI durability across all four pulmonary veins. This reinforces the hypothesis that pulmonary vein angulation is one determinant of ablation success and suggests that preprocedural CTA assessment of these parameters could help in procedural planning. Identifying patients with unfavorable PV anatomy may enable personalized ablation strategies, such as adjusting the transseptal puncture site, optimizing cryoballoon positioning, or considering alternative ablation strategies.

### Future directions of PV topography in PVI

Our study suggests that transseptal puncture planning should be tailored to individual PV topography to optimize cryoballoon PVI outcomes. Specifically, we found that superior PVs with a septum angle *α* of ≤73°(RSPV) and ≤117° (LSPV) were associated with higher rates of procedural success, indicating that the transseptal puncture site directly influences catheter alignment and complete PV isolation. A smaller vector angle *α* means that the septum and PV have a steeper relationship. A larger angle (>73° and >117°) means that both structures are positioned more flatly relative to each other. Following our results, a steeper (smaller) septum-to-vein angle seems to facilitate better cryoballoon positioning, ensuring more effective and durable pulmonary vein isolation. This position is achieved by a more inferior–posterior transseptal puncture. An optimal puncture location may help achieve better coaxial positioning of the cryoballoon, ensuring uniform tissue contact and complete circumferential ablation. Preprocedural CTA can be utilized to assess PV orientation and guide precise transseptal puncture positioning, potentially reducing procedural complexity and improving long-term PVI success rates. Future studies should validate these findings and establish standardized CTA-based criteria for individualized transseptal puncture planning, especially as exact guidance of the site of transseptal puncture becomes increasingly available with the emerging use of intracardiac echocardiography (ICE) in the context of reducing interventional radiation exposure.

PFA is an emerging technology in the electrophysiology field. Unlike cryoballoon ablation, which relies on thermal energy, PFA operates through nonthermal electroporation, targeting cardiac tissues while sparing nearby structures such as the esophagus and phrenic nerves. Recent studies have highlighted PFA's efficacy and safety. These findings underscore the promise of PFA as an alternative modality to cryoballoon ablation in managing paroxysmal AF. However, one might speculate that challenging PFA catheter positioning could occur as a consequence of PV topography and also influence procedural success. Therefore, future studies are needed to investigate the use of CTA-derived PV topography for dedicated PFA devices.

In summary, our findings suggest that preprocedural CTA parameters may provide insights into patient selection for PVI, potentially guiding the choice between cryoballoon and RF ablation based on anatomical features such as the angles between the septum, the PV, and the course of the PV. While parameters such as diameter, perimeter, area, and eccentricity did not influence 1-year freedom from AF recurrence, septum angle *α*, orifice angle *β*, and intra-atrial angle *γ* were associated with procedural success. However, our results should be interpreted as hypothesis-generating. However, large-scale prospective clinical trials are needed to prove the results of this pivotal feasibility study.

## Study limitations

This study has potential limitations. It is a retrospective study of two German centers and has enrolled a limited number of patients with paroxysmal AF and CTA prior to PVI. Furthermore, the study has a limited follow-up period and lacks a control group comparing the approach to other PVI techniques. Performing a preprocedural CTA is not standard of care in many centers and necessitates additional resources, expertise, and time for planning, which could limit widespread adoption. Furthermore, the use of CTA introduces additional radiation exposure and contrast agents, posing potential risks, particularly for patients with chronic kidney disease or those requiring multiple imaging studies. These factors may impact the practicality and cost-effectiveness of implementing this tool in routine clinical practice, necessitating further evaluation in prospective, multicenter trials. Selection bias might have influenced the results since only patients with CTA prior to PVI were included. Although clinical recurrence of AF is often attributed to PV reconnection, recurrence can also occur despite durable PV isolation, and not all patients with PV reconnection experience clinical recurrence. This limitation underscores the complexity of AF mechanisms beyond PV reconnection, which should be considered when interpreting the findings. Furthermore, occlusion grading using a contrast agent is limited by two-dimensional visualization of the occluded PV, and echocardiographic occlusion assessment by ICE as proposed in recent trials might help quantify and refine the PV occlusion ( 24 ). Another limitation of our study is the retrospective determination of mapping catheter placement in early branching PVs, as our CTA-based angle measurements were applied to the specific branch where the catheter was positioned. This approach, while ensuring accurate postprocedural correlation, does not directly allow for prospective prediction of the optimal branch for catheter placement. In clinical practice, procedural factors such as anatomical accessibility, branch angulation, and operator preference may influence branch selection. Future studies should aim to validate whether preprocedural CTA angles correlate with the actual procedural branch chosen, potentially aiding preprocedural planning and patient selection.

The sphericity index may be a better parameter for assessing complex PV anatomy, as it accounts for irregular contours and variations in shape beyond simple elongation. However, eccentricity is more useful when considering only the relative elongation of the PV ostium but may not capture irregular shapes or lobulations. However, the software used in our study did not obtain the sphericity index, so these data are lacking.

Therefore, the results should be regarded as hypothesis-generating and need to be supported by larger-scale randomized, multicenter trials.

## Conclusions

Patient selection using CTA prior to cryoballoon ablation might influence the procedural success of cryoballoon PVI. While PV anatomy in regard to vein size and shape was not associated with procedural outcome, septum, orifice, and intra-atrial angulations were related to procedural success. Identifying unsuitable PV topography in preprocedural CTA might help to improve the procedural success rate of PVI using balloon technologies.

## Data Availability

The datasets generated and/or analyzed during the current study are not publicly available due to data protection and privacy regulations, as they contain sensitive patient information. Access to the data is therefore restricted and subject to institutional and ethical approvals. Requests for access to the data should be directed to the corresponding author.
