# Supplementary figures and images for: CT angiography-derived three-dimensional pulmonary vein topography is related to the outcome after cryoballoon ablation
Source: Front Cardiovasc Med. 2025 May 8;12:1496922. doi: 10.3389/fcvm.2025.1496922 (PMC12096455; doi:10.3389/fcvm.2025.1496922)

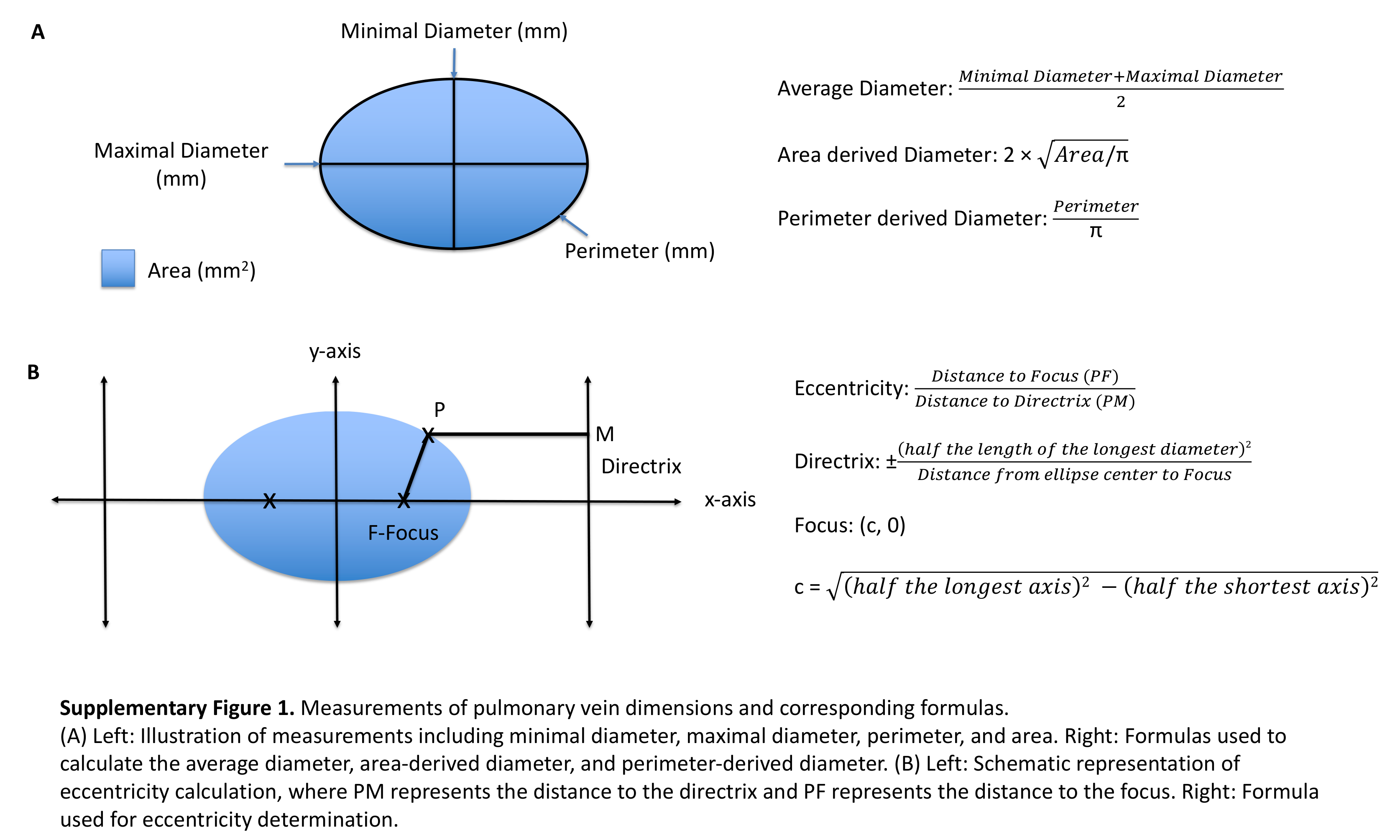

Supplement: Supplementary file 1 [file Image1.tiff]

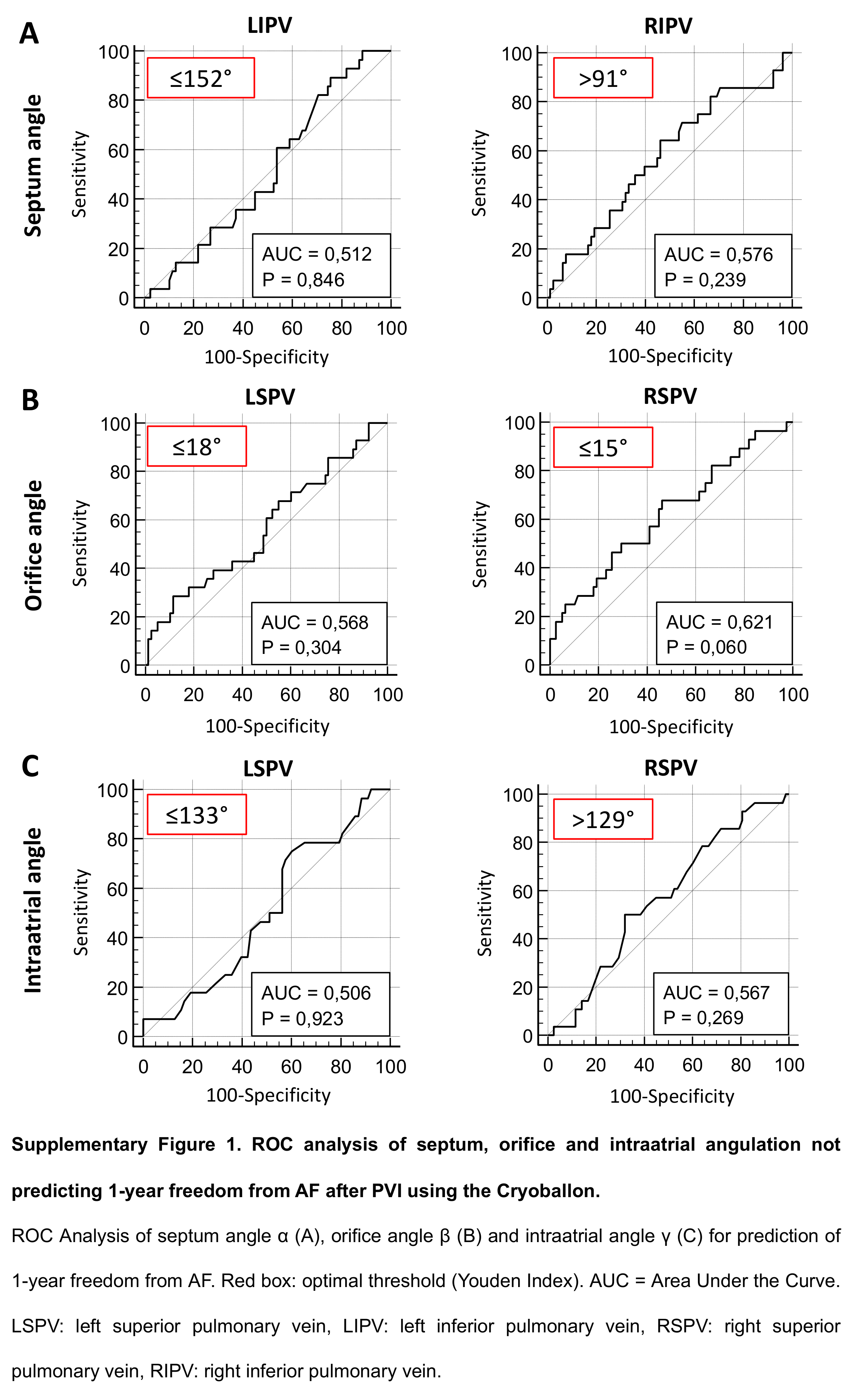

Supplement: Supplementary file 2 [file Image2.tiff]
